# Investigating *TSHR* gene variants in consanguineous families: novel insights into variable expression in familial congenital hypothyroidism

**DOI:** 10.3389/fendo.2025.1559281

**Published:** 2025-05-05

**Authors:** Zakiye Nadeali, Zohreh Mohammadi-Zaniani, Sajjad Biglari, Newsha Molavi, Khashayar Zardoui, Sam Mirfendereski, Mahin Hashemipour, Mohammad Amin Tabatabaiefar, Constantin Polychronakos

**Affiliations:** ^1^ Department of Genetics and Molecular Biology, School of Medicine, Isfahan University of Medical Sciences, Isfahan, Iran; ^2^ Montreal Children’s Hospital and the Endocrine Genetics Laboratory, Child Health and Human Development Program, Research Institute of the McGill University Health Centre, Montreal, QC, Canada; ^3^ Immunodeficiency Diseases Research Center, Isfahan University of Medical Sciences, Isfahan, Iran; ^4^ Department of Radiology, Isfahan University of Medical Sciences, Isfahan, Iran; ^5^ Metabolic Liver Disease Research Center, Isfahan University of Medical Sciences, Isfahan, Iran; ^6^ Child Growth and Development Research Center, Research Institute for Primordial Prevention of Non-Communicable Disease, Isfahan University of Medical Sciences, Isfahan, Iran

**Keywords:** congenital hypothyroidism, thyroid-stimulating hormone receptor, exome sequencing, consanguineous, familial

## Abstract

**Background:**

A defective thyroid-stimulating hormone receptor (*TSHR*) gene is one of the main known genetic factors leading to congenital hypothyroidism (CH). However, the relationship between TSHR genotypes and phenotype and the underlying reason for the broad spectrum of phenotypes in the patients carrying *TSHR* gene defects have not yet been clearly established. This study aimed to investigate the genetics of patients with CH to identify *TSHR* defects and to explore the specific extrathyroidal defects and other phenotypic features in these patients to establish a genotype-phenotype correlation.

**Methods:**

Consanguineous families with primary CH and a history of non-autoimmune acquired hypothyroidism were included in this study. The causative variants in the *TSHR* gene were identified using exome sequencing. Multiple *in silico* analysis tools were employed to interpret the variants.

**Results:**

Five *TSHR* variants including two novel variants were identified in patients with thyroid dysgenesis from five families. Some patients presented inter- and intra-familial variable expression and different ages of onset. The data suggest the possibility that the clinical phenotype of patients with CH caused by *TSHR* variants can be influenced by the coexistence of other gene defects.

**Conclusions:**

This study investigated the variants of the *TSHR* gene contributing to CH for the first time in Iran. Our study on multiplex consanguineous families could help provide further evidence for the elucidation of the oligogenic inheritance in CH, possibly leading to variable expressivity in patients with CH. These data could have implications for genetic diagnosis and counseling to identify deleterious variants for possible diagnostics, clinical management, and preventive aims.

## Introduction

Congenital hypothyroidism (CH), the inborn condition of thyroid hormone deficiency, is a common endocrine disorder in neonates. Delayed diagnosis and treatment of CH can result in irreversible neurological problems (1). While the worldwide incidence of CH ranges from 1:2500 to 1:3000 live births, the incidence in Iran is 1:370 to 1:1433 (2, 3).

In the majority of affected infants, CH is permanent, necessitating lifelong levothyroxine (L-T4), and results from abnormal thyroid gland development [thyroid dysgenesis (TD)] or a defect in thyroid hormone synthesis [thyroid dyshormonogenesis (DH)] (4). In rare cases, the disorder may be of central origin and due to an intrinsic defect of the hypothalamic-pituitary-thyroid axis. However, in some patients, factors such as maternal antibodies or iodine insufficiency cause a transient thyroid dysfunction that resolves over time (5, 6).

The thyroid-stimulating hormone receptor (TSHR) plays a crucial role in the regulation of thyroid function, thus contributing as a key genetic factor to the pathogenesis of CH. TSHR belongs to the G-protein-coupled receptors (GPCR) family and is located on the basement membrane of the thyroid follicle. Activated upon binding to TSH, TSHR mediates the TSH functions to control thyrocyte differentiation and proliferation and promotes the synthesis and secretion of thyroid hormones. It has a central role in regulating the development and function of the thyroid gland (7–9).

Loss-of-function (LOF) variants in the thyroid stimulating hormone receptor (*TSHR*) gene are associated with TSH resistance, a hallmark of congenital non-goitrous hypothyroidism. In this condition, thyroid follicular cells have reduced sensitivity to stimulation by TSH, leading to a broad spectrum of phenotypes ranging from profound thyroid hypoplasia due to poor thyroid differentiation (thyroid dysgenesis) to mild subclinical CH with a thyroid gland of normal size. In contrast, gain-of-function variants can lead to dominantly transmitted hyperthyroidism (6, 9–11).

The severity of the disease related to these variants depends on the degree of the impairment of the TSHR function resulting from dominant (monoallelic) or recessive (biallelic) inheritance of the variants (12). *TSHR* monoallelic variants have been reported to contribute to a mild phenotype whereas biallelic variants in the *TSHR* gene result in a more severe phenotype (13). Moreover, patients with presumed oligogenic inheritance with variants in other CH-related genes, in addition to TSHR, have been reported (14).

*TSHR* gene mutation was first described in 1995 (OMIM 603372) (15). More than 264 different TSHR mutations have been described in the *TSHR* gene in the Human Gene Mutation Database (HGMD) as of November 2024 (http://www.hgmd.org). The reported incidence of the pathogenic variants in *TSHR* in CH differs, ranging from 0% to 30.6% (12, 14, 16–18). Moreover, homozygous *TSHR* variants are reported as a frequent cause of CH with a family background in different studies (13, 19).

However, the genotype-phenotype relationships have not yet been clearly established. Patients with CH are at a higher risk of developing extrathyroidal defects compared to the general population (20). Therefore, genetic testing in newborns with CH could contribute to the identification of the responsible gene, leading to the early detection of these abnormalities and better patient care and prevention of late complications.

This report outlines the clinical, biochemical, and genetic profiles of patients with CH harboring *TSHR* variants in Iran. Through a detailed clinical examination, we explored potential extrathyroidal defects associated with the *TSHR* variants and defined the genotype-phenotype correlation. This study helps expand the mutational and clinical knowledge on the *TSHR* gene and offers additional insights into understanding the genetic etiology of primary CH and clinical management in patients carrying *TSHR* variants in a society where consanguineous unions are not uncommon.

## Materials and methods

### Family recruitment and study design

Families with multiple affected members with non-autoimmune hypothyroidism were referred to the Isfahan Endocrine and Metabolism Research Center, Isfahan University of Medical Sciences, Iran between 2004 and 2021. The patients included in this study were those with CH carrying TSHR variants, as tested by exome sequencing. All patients were of Iranian descent.

CH was diagnosed based on elevated serum thyroid-stimulating hormone levels (TSH; ≥10 mIU/L) and decreased free thyroxine (FT4; <0.8 ng/dl). The patients were either diagnosed through a newborn screening (NBS) program (heel blood TSH, ≥9.0 mIU/L) (six patients) or diagnosed later in life after hypothyroidism manifestations (two patients). Thyroid morphology was determined using ultrasonography (US) examinations to differentiate between TD and DH. All patients required continued administration of L-T4 for more than 3 years, confirming the disease’s permanent status. The presence of autoimmune hypothyroidism in patients was excluded by measuring serum levels of anti-thyroid peroxidase (anti-TPO) and anti-thyroglobulin (anti-TG) antibodies.

The study design and protocol were approved by the Ethics Committee of Isfahan University of Medical Sciences (approval no. IR.MUI.MED.REC.1401.183) and McGill University (approval no. 2024-9933). Written informed consent was provided by the participants’ legal guardians. Medical history was obtained via genetic counseling, and the pedigree was drawn using the “Progeny” software (Progeny Software, LLC).

### Molecular genetic studies

Peripheral blood was taken, and DNA extraction was conducted using the CTGA Ultrarapid DNA extraction kit provided by CinnaTeb Gen Azma Co., Iran.

Exome sequencing (ES) was performed at the Genome Québec Centre, Montreal, Canada and results were analyzed and interpreted at the Research Institute of the McGill University Health Center. Briefly, DNA libraries were constructed using an Agilent SureSelect V6 enrichment capture kit (*Agilent* Technologies, Inc., Woburn, MA, USA), and the captured library was sequenced with NovaSeq6000 (*Illumina*, Genome Québec, Montreal, Canada). Read alignment and mapping to GRCh38 and variant calling were conducted using DRAGEN v3.9.5 (Illumina).

### Calling of TSHR variants from exome data

The identified single nucleotide variants (SNVs), Multi-nucleotide variants (MNVs), and insertion/deletion (InDels) were annotated using ANNOVAR and NIRVANA Variant Annotator (*Illumina*). For filtration and prioritization of variations, we used locally developed pipelines. Variants with a minor allele frequency (MAF) <0.01 in the gnomAD (gnomad.broadinstitute.org) database were selected for further analysis. SNVs were evaluated using the *in silico* meta-predictors Rare Exome Variant Ensemble Learner (REVEL) (21) and Combined Annotation Dependent Depletion (CADD) (22). Variants with a REVEL score > 0.5 (23) or CADD-Phred score >17.74, based on a mutation significance cutoff (MSC) of 17.74 for TSHR (24) were further assessed using MutationTaster to determine whether they were disease-causing (mutationtaster.org).

The variants were classified based on the American College of Medical Genetics and Genomics (ACMG) guidelines (25). The novelty of these variants was investigated in the HGMD (http://www.hgmd.cf.ac.uk/ac/index.php), CLINVAR database, and relevant literature. The variants detected by ES were confirmed by Sanger sequencing using standard PCR amplicons to assess co-segregation patterns in the pedigree. The related family history and previous medical records were obtained through genetic counseling.

### *In silico* analysis and homology modeling

Nonsense-mediated decay (NMD) prediction of the c.1426_1432 del variant was performed using the NMDEsc Predictor server to predict whether this frameshifting will lead to NMD+ or NMD− transcripts based on the relative location of the variant within the *TSHR* gene.

In addition, the degree of conservation of the novel missense variant was assessed in several species using MEGA6 software (26).

Homology modeling was performed using the Alpha fold Protein Structure Database with the code AF-P16473-F1-v4. PyMOL (Version 2.2.3, Schrödinger, LLC.) software was used for visualization, mutagenesis, and structural analysis. Furthermore, multiple stability prediction tools (SAAFEC-SEQ, mCSM, INPS-3D, and I-Mutant2.0) were used to evaluate the effects of mutations on the protein stabilities.

### Homozygosity mapping

Due to the consanguineous nature of the families, we used the Automap tool (Autozygosity Mapper, Basel, Switzerland), v1.2, to identify the runs of homozygosity (ROH) via the command-line package using default parameters and hg38 as the reference genome on Variant Call Format (VCF) calls (27). After receiving the output files, variants identified by ES were further filtered according to the ROH regions. ROH scores >90% of the maximum and > 0.5 Mb in size were investigated further. All regions with >0.5 Mb of homozygosity shared by affected individuals were considered candidate regions for the phenotype. The files generated included the predicted ROHs and were used afterwards for variant prioritization in consanguineous cases.

## Results

### Clinical and biochemical characteristics

Among families with primary patients with CH referred to our center, five consanguineous families with multiple affected individuals with non-autoimmune hypothyroidism carried inactivating *TSHR* variants. **Table 1** presents detailed biochemical and clinical characteristics of the patients as well as thyroid functional information. Based on the serum FT4 level at diagnosis, CH was categorized as severe [FT_4_ <5 pmol/L (<0.39 ng/dL)], moderate [FT_4_ 5–<10 pmol/L (0.39–<0.78 ng/dL)], or mild [FT_4_ ≥10 (≥0.78 ng/dL)] (28).

**Table 1:** Clinical and biochemical data of the patients with childhood-onset hypothyroidism.

Patient ID	Sex	Age at diagnosis	TSH at diagnosis (mIU/L)	FT4 at diagnosis (pmol/L)	Extrathyroidal abnormalities	Current LT-4 dose (mg/day)	Thyroid ultrasound	Severity classification (mild/moderate/severe)	Variants	Maternal		Paternal	
										Genotype	Phenotype Thyroid morphology/LT4 dose	Genotype	Phenotype Thyroid morphology/LT4 dose
1a	Male	Through NBS	64	8.9	Umbilical hernia, varicocele, strabismus, learning difficulty	143	Athyreosis	Moderate	*TSHR*:c.484C>T/ *SLC26A7*: c.974C>A	*TSHR*: Mut/WT/ *SLC26A7*: Mut/WT	Hypoplasia/100	*TSHR*: Mut/WT	Normal/NA
1b	Female	5.5 years	28	8.7	–	100	Hypoplasia	Moderate	*TSHR*:c.484C>T				
53a	Male	15 days	18.24	0.63	Ophthalmic manifestations	150	Athyreosis	Severe	*TSHR*:c.224T>C	Mut/WT	Normal/NA	Mut/WT	Normal/NA
53b	Male	30 days	66.93	0.77	Ophthalmic manifestations	150	Athyreosis	Severe	*TSHR*:c.224T>C				
11	Male	Through NBS	11.7	9.8	–	42.8	Hypoplasia	Moderate	*TSHR*:c.1426_1432del	WT/WT	Normal/NA	Mut/WT	Hypoplasia/100
38	Female	Through NBS	51	5.9	–	64.2	Hypoplasia	Moderate	*TSHR*:c.484C>G/ c.1591C>T	Mut/WT	Hypoplasia/71.5	Mut/WT	Normal/NA
41a	Male	Through NBS	28.2	6	Undescended testis, KAT6B-related manifestations	107	Hypoplasia	Moderate	*TSHR*:c.484C>G/ *KAT6B*: c.4682A>C	*TSHR*: Mut/WT/ *KAT6B*:Mut/WT	Hypoplasia/121.5	*TSHR*: Mut/WT	Normal/NA
41b	Female	6 years	11	7.7		107	Hypoplasia	Moderate	*TSHR*:c.484C>G				

NBS, newborn screening; TSH, thyroid-stimulating hormone.

**Figure 1:**
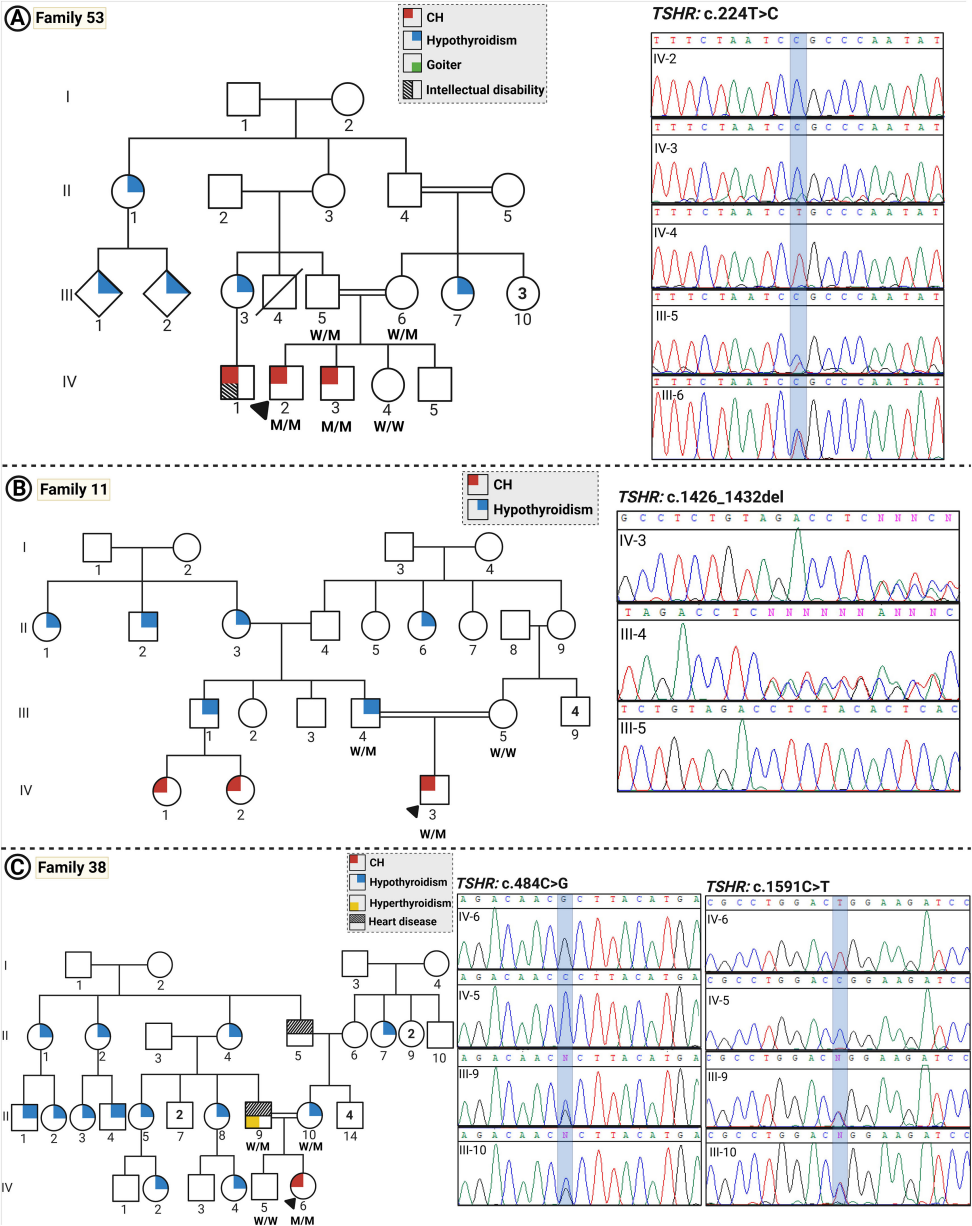
The pedigrees and Sanger sequencing of families with CH. **(A–C)** show pedigrees and the Sanger sequencing data for families 53, 11, and 38, respectively. Individuals diagnosed with childhood-onset hypothyroidism are represented by gray solid-filled symbols, whereas white symbols indicate individuals with subclinical hypothyroidism diagnosed in adulthood and normal thyroid function, respectively. (Squares and circles represent males and females, respectively; a diagonal line indicates deceased subjects; consanguineous parents are determined via a double horizontal line; the proband is arrowed).

**Figure 2:**
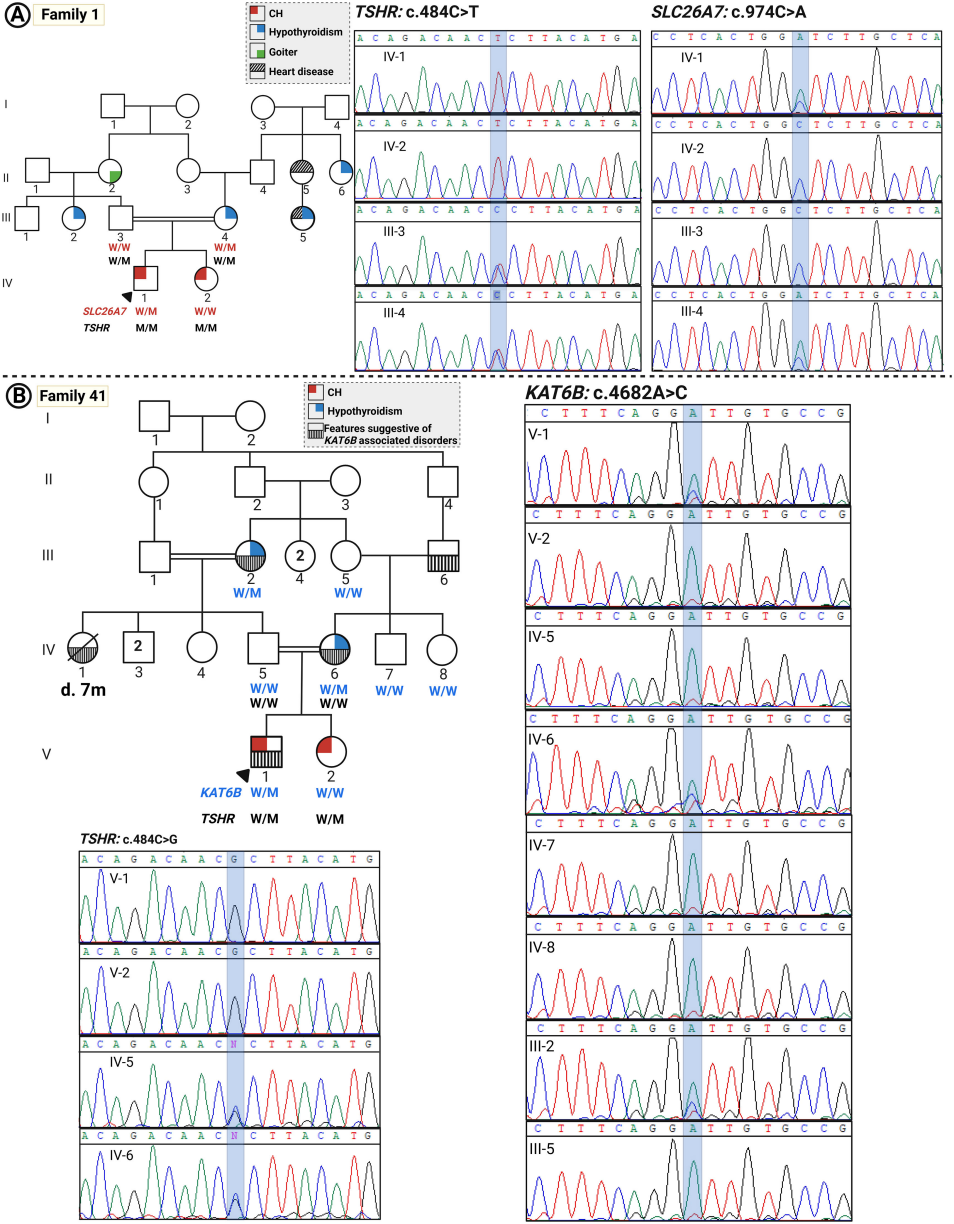
The pedigrees of families with CH with oligogenic inheritance. **(A)** Pedigree of family 1 and the related Sanger sequencing data indicating the co-segregation of the variants in the *TSHR* and *SLC26A7* genes with disease. **(B)** Pedigree of family 41 and co-segregation data of variants in the *TSHR* and *KAT6B* genes.

Detected variants were assessed using literature searches, dbSNP (database of SNP) or gnomAD (Genome Aggregation Database, v4.1.0), Iranome (http://www.iranome.ir/) for population allele frequency analysis, and ClinVar database. New variants were assessed *in silico* for pathogenicity using REVEL and CADD and were classified based on ACMG guidelines. Genotypic data and inheritance for the *TSHR* variant in five families are presented in **Table 2**. An overview of the TSHR variants and their co-segregation in each pedigree are shown in **Figures 1**, **2**.

**Table 2:** Detailed Genotyping data of the detected variants in relation to CH phenotype.

Family ID	Nucleotide Change	Amino Acid Change	Mutation Type	rs ID	Zygosity	gnomAD v.4.1.0 MAF	ClinVar	Mutation Taster	REVEL Score	CADD score	ACMG classification	Reference
F1	TSHR:c.484C>T	p.Pro162Ser	Missense	rs121908863	Homo	NA	VUS	Deleterious	0.44	24.4	P (PM5, PM1, PM2, PP1, PP4, PM3)	(29)
	SLC26A7: c.974C>A	p.Ala325Asp	Missense	NA	Het	NA	NA	Deleterious	0.73	27.8	LP (PM2, PP3, PP1)	NA
F53	TSHR:c.224T>C	p.Leu75Pro	Missense	NA	Homo	NA	NA	Deleterious	0.86	27.3	LP (PM3, PM2, PP3, PP4, PP2)	NA
F11	TSHR:c.1426_1432del	p.Tyr476ThrfsTer35	Deletion		Het	NA	NA	NA	NA	NA	P (PVS1, PM2, PP4, PP1)	NA
F38	TSHR:c.484C>G	p.Pro162Ala	Missense	rs121908863	Homo	0.0002	P/LP	Deleterious	0.46	23	P (PP5, PM1, PM5, PM2, PP2)	(29–32)
	TSHR:c.1591C>T	p.Arg531Trp	Missense	rs139892516	Homo	0.00006	VUS	Deleterious	0.56	24.1	VUS (PM2, PP3, PP2, PP4)	(16, 33–35)
F41	TSHR:c.484C>G	p.Pro162Ala	Missense	rs121908863	Homo	0.0002	P/LP	Deleterious	0.46	23	P (PP5, PM1, PM5, PM2, PP2)	(29–32)
	KAT6B:c.4682A>C	p.Asp1561Ala	Missense	NA	Het	NA	NA	Deleterious	0.544	24.3	VUS (PM2, PP4, PP1)	NA

PM5: another mutation that causes the same amino acid change previously reported as pathogenic; PM1: located in a mutational hot spot without benign variation; PM2: absent from controls in Exome Sequencing Project, 1000 Genomes Project, Exome Aggregation Consortium, and Iranome (local database); PP1:Co-segregating in the family; PP3: multiple lines of computational evidence support a deleterious effect on the TSHR protein; PP4: Patient's phenotype or family history is highly specific for a disease with a single genetic etiology; PP5: reputable source recently reports variant as pathogenic, but the evidence is not available; PM3: For recessive disorders, detected in a homozygous state in affected case; PP2: Missense variant in a gene in which missense variants are a common mechanism of disease.

Homo, homozygous; Het, heterozygous; P, pathogenic; LP, likely pathogenic; VUS, variant of uncertain significance; NA, not applicable.

**Figure 3:**
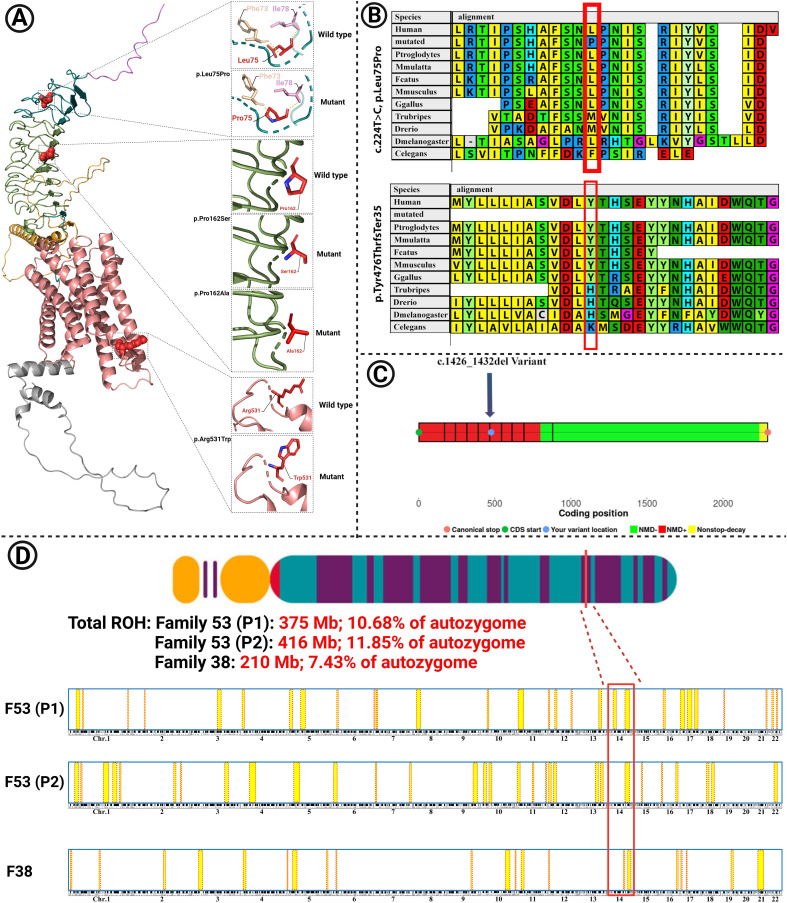
**(A)** The modeled structure of the mutated TSHR protein containing the p.Leu75Pro, p.Pro162Ser, p.Pro162Ala, and p.Arg531Trp variants. Domains and colors: (Backbone: Deep teal): 1. SP (signal peptide): 1–20, violet; 2. LRR: 100–271, smudge; 3. Hinge region: 280–410, bright orange; 4. TMD (transmembrane) domain: 414–682, salmon; 5. ICD (intracytoplasmic) domain: 683–764, gray. **(B)** The variants p.Leu75Pro and p.Tyr476ThrfsTer35 are located in highly conserved regions among different species. **(C)** Nonsense-mediated decay (NMD) prediction of the c.1426_1432 del variant in the *TSHR* gene using the NMDEsc predictor server. The variant was located in the red-colored regions, and it is predicted to be targeted for NMD. Green-colored regions are not targeted for NMD. **(D)** Homozygosity mapping in families 53 and 38. The shared homozygosity region in families 53 (P1 and P2) and 38 is depicted. Total runs of homozygosity (ROH) are also shown for each patient.

**Figure 4:**
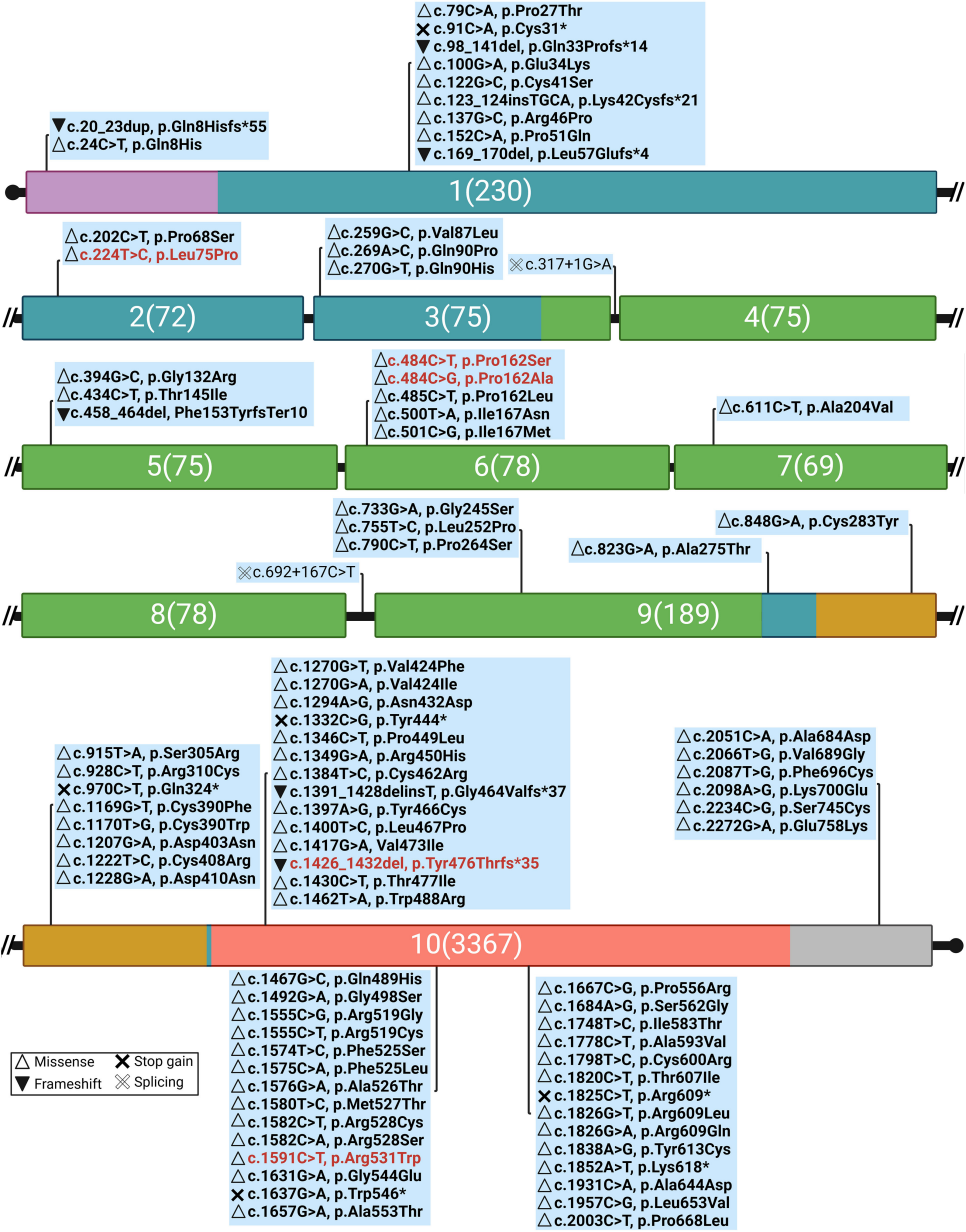
Genomic distribution of CH-related variants along *TSHR* exons, highlighting novel variants in this study (red text) and their effects on protein function [Mutation Database (accessed on 5 February 2025, https://www.tsh-receptor-mutation-database.org/). Domains and colors (Backbone: Deep teal): signal peptide (SP), violet; leucine-rich repeats (LRR), smudge; hinge region, bright orange; transmembrane (TMD) domain, salmon; intracytoplasmic (ICD) domain, gray. The numbers: the exon number followed by the number of nucleotides.

Five different *TSHR* gene variants were detected in these patients, two of which are novel: c.224T>C (p.Leu75Pro) and c.1426_1432del (p.Tyr476ThrfsTer35) (**Figure 3**, **Table 2**), while three have been published previously (c.484C>T, c.484C>G, and c.1591C>T). **Figure 4** summarizes known variants on the *TSHR* gene that cause CH, along with the newly identified variants in this study.

### Family 53 (two patients)

This was a consanguineous family (first cousin parents) with an autosomal recessive pattern of inheritance with four children, two of which were affected boys, and both parents and two other children (a girl and a boy) were healthy as assessed by thyroid ultrasound and biochemical examinations.

Patient 1 (P1) was a 23-year-old male and patient 2 (P2) was a 19-year-old male with athyreosis as tested by thyroid ultrasound examination. Both affected siblings had a clinical history of ophthalmic abnormalities such as persistent strabismus and light sensitivity. Both parents had normal thyroid function.

The two affected siblings were homozygous for a novel missense variant in the *TSHR* gene (NM_000369.5, c.224T>C, p.Leu75Pro). Sanger sequencing confirmed the segregation of this variant with the CH phenotype in the family. According to ACMG guidelines, the c.224T>C variant is classified as likely pathogenic (**Table 2**, **Figure 1**).

### Family 11 (one patient)

The proband was born to first cousin parents. He demonstrated intermediate hypothyroidism in terms of severity, with thyroid hypoplasia. The father was also hypothyroid with a hypoplastic gland, and the mother had autoimmune hypothyroidism.

A heterozygous frameshift deletion in the *TSHR* gene [NM_000369.5, c.1426_1432del, p.(Tyr476ThrfsTer35)] was detected in the proband which co-segregated with the thyroid disease in the family and showed the inheritance of the disease from the hypothyroid father. Based on ACMG guidelines, the c.1426_1432del variant is classified as pathogenic.

### Family 38 (one patient)

This was a highly multiplex consanguineous family (first cousin parents) with a daughter affected with CH, a hypothyroid mother, and a hyperthyroid father. In terms of severity, she presented with moderate CH. Born to a highly multiplex family, two homozygous variants in *TSHR* c.484C>G (NM_000369.5:c.484C>G, p.(Pro162Ala)) and 1591C>T (NM_000369.5:c.1591C>T, p.(Arg531Trp) were detected in the proband. Both variants have been reported previously to cause CH. These variants were inherited in a homozygous state in the affected individual for which her parents were heterozygous, confirming the segregation of the mutation with CH in the family. The variant c.484C>G is pathogenic according to the ACMG guideline criteria. Variant 1591C>T is of uncertain significance based on the ACMG guideline.

### Family 1 (two patients)

Two children with CH were born to consanguineous parents (first cousins); patient 1 (P1) was a 22-year-old male diagnosed through the NBS program. He exhibited umbilical hernia and strabismus. His US examination demonstrated an apparent athyreosis (severe hypoplasia). His genetic analysis revealed a homozygous missense variant in the *TSHR* gene (NM_000061.3:c.484C>T; p.Pro162Ser). However, patient 2 (P2) who did not undergo NBS, was diagnosed at the age of 5 years. Her thyroid gland size was slightly reduced as tested by US, and she is mentally and cognitively normal. Their mother’s thyroid, who was on levothyroxine treatment, was also hypoplastic. Their father showed sub-clinical hypothyroidism with a normal-sized thyroid gland. P2 also showed the same homozygous *TSHR* mutation on genetic analysis as her brother. Both parents were heterozygous for this variant (**Figure 2**). According to the ACMG guideline, this variant is classified as a likely pathogenic variant.

The affected male patient and the hypothyroid mother also carry a heterozygous variant in the *SLC26A7* gene (NM_052832.4:c.974C>A p.(Ala325Asp) which is a known CH-related gene (36, 37). This variant could explain the more severe phenotype in the proband and the carrier mother, compared to the family members with the same genotype, i.e., P2 and the father with sub-clinical hypothyroidism, respectively. According to the ACMG guidelines, this variant is classified as a likely pathogenic variant.

### Family 41 (two patients)

Patient 1 (P1) was a 13-year-old male, the offspring of first-cousin parents diagnosed through NBS with a clinical history of undescended testis. However, his older sister [patient 2 (P2)], aged 22 years, was diagnosed with hypothyroidism at the age of 6 years. Thyroid ultrasound demonstrated thyroid hypoplasia in both siblings affected with CH and their hypothyroid mother. The father was euthyroid.

The proband was homozygous for c.484C>G; p.Pro162Ser in the *TSHR* gene, which was also reported in family 38.

Another variant identified in this family was *KAT6B* (NM_012330.4):c.4682A>C p.(Asp1561Ala). Many affected individuals with *KAT6B* mutations have been reported to present abnormalities of thyroid structure or function as a part of a broad clinical spectrum called KAT6B disorders, the variable expressivity of which is increasingly being recognized. The heterozygous variants of the *KAT6B* gene are related to KAT6B disorders with a highly variable expressivity, in which functional or structural thyroid abnormalities such as hypothyroidism and thyroid agenesis or hypoplasia are present in many affected individuals (38). The clinical history of the patients included respiratory disease (central or obstructive sleep apnea) in the proband; cardiovascular disease in the mother that presented at the age of 35 years, growth anomalies including growth delay and poor weight gain in the proband; long thumbs and abnormalities of the toes such as great toes and overlapping toes in the proband, his mother, and maternal grandfather; undescended testis in the proband; multicystic dysplastic kidneys in the mother and maternal grandfather; distinctive facial appearance in the mother; ocular anomalies in the proband and his mother; language disorders and speech difficulties; dental anomalies; and history of other features such as scoliosis in the family, suggestive of the presence of a KAT6B disorder subtype with variable expression in the family members harboring this variant. This variant is a variant of uncertain significance (VUS) according to the ACMG guideline.

### Homology modeling

The variant c.1426_1432 del, which was identified in family 11, is predicted to cause the protein to undergo nonsense-mediated decay (**Figure 3C**).

Homology modeling for the missense variants showed that p.Leu75Pro in TSHR (Family 53), has an alpha helix secondary structure and is located in the TSHR extracellular domain (amino acids 1–260). This variant is highly conserved among several species, including *M. musculus, G. gallus, T. rubripes*, and *D. melanogaster*, as assessed using MEGA6 software (**Figure 3A**). The identified missense variants are destabilizing according to the protein stability prediction tools (**Table 3**). The substitution of proline 162 to serin, which was identified in family 1, and to alanine in families 38 and 41, is located at the border between the beta strand and the segment connecting it to the next alpha helix and is situated in the TSHR extracellular domain.

**Table 3:** The prediction server results for protein stability due to our deleterious variants.

Stability prediction server	INPS-3D	mCSM	DUET	SDM	SAAFEC-SEQ	I-Mutant2.0
**p.Leu75Pro**	Destabilizing	Destabilizing	Destabilizing	Destabilizing	Destabilizing	Decreased stability
**p.Pro162Ser**	Destabilizing	Destabilizing	Destabilizing	Destabilizing	Destabilizing	Decreased stability
**p.Pro162Ala**	Destabilizing	Destabilizing	Destabilizing	Stabilizing	Destabilizing	Decreased stability
**p.Arg531Trp**	Destabilizing	Destabilizing	Destabilizing	Destabilizing	Destabilizing	Decreased stability

“INPS-3D”, “mCSM”, “DUET”, “SDM”, and “SAAFEC-SEQ are protein stability prediction tools. INPS-MD, Impact of Non-synonymous mutations on Protein Stability - Multi Dimension; mCSM, mutation Cutoff Scanning Matrix; DUET, Dual Energy Transformation; SDM, Site Directed Mutator; SAAFEC-SEQ, Sequence-baesd Single Amino Acid Folding free Energy Changes (SAAFEC) method.

### Homozygosity mapping

Due to familial consanguinity in the pedigrees, the ES data were used for SNV-based homozygosity mapping (HM). The shared homozygosity region for the patients in family 53 (P1 and P2) and in family 38 were 28.63Mb and 11.36Mb, respectively. The total ROH in the probands of family 1 and family 2 were 375, 416, and 210 Mb, respectively (10.68%, 11.85%, and 7.43% of the autozygome), consistent with the presence of extensive consanguinity (**Figure 3D**).

## Discussion

A defective *TSHR* gene is one of the main genetic factors leading to CH. This study was the first to analyze the *TSHR* mutation spectrum of patients with CH in Iran. All the patients included in this study were born to consanguineous parents and had a positive family history. They showed moderate to severe hypothyroidism, with a hypoplastic thyroid gland or athyreosis (thyroid dysgenesis). Through comprehensive screening, we identified five TSHR variants as the cause of CH in five consanguineous families with primary permanent CH from Iran. Exome sequencing and subsequent PCR-based Sanger sequencing revealed five variants, two of which are novel (c.224T>C and c.1426_1432del) but three variants (c.484C>T, c.484C>G and c.1591C>T) have previously been reported.

The *TSHR* gene on chromosome 14q3 consists of 10 exons and codes for a 764-aa protein, which includes a signal peptide of 21 aa, a large ectodomain of 394 residues (extracellular region forming the amino terminus) encoded by nine exons which form the binding domain for TSH, and 349 residues encoded by the tenth and largest exon, which constitute the seven transmembrane segments (the carboxyl-terminal region that forms the intracytoplasmic domain). These transmembrane segments are joined intracellularly through connecting loops that interact with G proteins upon activation of the receptor (39, 40). The ectodomains contain two cysteine clusters flanking nine leucine-rich repeats (LRR). In the present study, the variants c.224T>C (Leu75Pro) and c.484C>G (Pro162Ala) c.484C>G (Pro162Ser) are located in the LRR domain and are expected to decrease the binding activity of TSH, subsequently leading to a reduction in cAMP production activities, according to the literature (41, 42). Although variant c.224T>C (Leu75Pro) caused a severe phenotype in both probands of family 53, the other variants in the LRR regions resulted in an intermediate phenotype in the patients (**Table 1**).

Moreover, the novel variant c.1426_1432del found in this study results in the elimination of the entire molecule and is predicted to cause NMD. Both the father and his affected son in family 11 were heterozygous for this variant, showing intermediate disease severity (**Table 1**).

Loss of function variants of *TSHR* cause TSH resistance in either the dominant or recessive mode of inheritance. The associated hypothyroid phenotype is variable depending on the type and location of the variants and how much TSHR function is affected, from euthyroid state and partial thyroid dysfunction (mild/borderline hypothyroidism) with a normal thyroid gland to severe CH with thyroid dysgenesis. Indeed, the mutation-specific damage to the functional and structural integrity of the receptor determines disease severity (43, 44).

In our study, three families carried a homozygous deleterious variant at position c.484, two of which with the c.484 C>G variant (families 38 and 41) and one with the c.484C>T variant (family 1). Functional studies on variant p.Pro162Ala have shown a partial decrease in function, which resulted in a mild permanent CH phenotype in homozygous patients and gestational hypothyroidism in a carrier mother (17). Interestingly, the patients carrying these variants in our study were inconsistent in terms of disease severity and age of onset. This was true even in patients within a family with identical *TSHR* genotypes. Phenotypic variability in patients with CH in a family has also been reported in previous studies (13, 43). The reason for the heterogeneous clinical presentation and different ages of onset could be attributed to other genes, maternal health, or environmental modulators (e.g. iodine sufficiency). Epigenetic mechanisms have also been proposed to contribute to CH (6).

Rare pathogenic variants that solely result in minor functional defects are supposedly associated with more severe phenotypes when co-inherited. The finding that the carrier phenotype of heterozygous parents had milder hypothyroidism later in life compared to the homozygous affected offspring further supports the hypothesis that the sum of mutations contributing to thyroid dysfunction could determine the disease manifestations in patients, which is inconsistent with the previous reports (12, 45). Importantly, evidence of oligogenic inheritance has been suggested as an explanation for the complex genotype-phenotype correlations in CH. Several studies have confirmed the oligogenicity in CH through co-segregation of variants of various combinations of CH genes with hypothyroid phenotype, one of which is *TSHR* (14, 46–50). Compelling evidence for digenic inheritance was found in a study in which 13 out of 45 cases with TSHR mutations, also had diallelic variants in DUOX2, although a formal mutation burden analysis was not presented (14). The present study is of additional value as we investigated the phenotype of the patients in multiplex families with an identical *TSHR* variant in a homozygous state and other CH-related gene defects more in-depth to test the reason for variable phenotypic expressions within a family. We also considered the age of onset, parental phenotype, LT4 doses, thyroid morphology, and extrathyroidal defects to provide a more comprehensive assessment of the patient’s condition. Detection of clinical complications in the patients in addition to FT4 and TSH at diagnosis in our study is important since even sub-clinical hypothyroidism can be associated with various diseases and it has been hypothesized that interpreting thyroid function on a continuous scale might be better than a binary interpretation based on fixed reference ranges (51). In the present study, the family members harboring both *TSHR* and other CH pathogenic genes (*SLC26A7* and *KAT6B)* presented with more complications and earlier onset compared to patients with the same *TSHR* variants. Notably, we observed this extensive variable expressivity only in two families with oligogenicity. Hence, we suggest that the coexistence of defects in other genes known to cause a hypothyroid phenotype with a defective *TSHR* gene might contribute to phenotype variability, different age of onset, and the presence of extrathyroidal defects between the family members with the same *TSHR* genotype. It is important to detect the reason for later onset hypothyroidism when monitoring the carriers and starting appropriate treatment. However, we did not find a reason for the different ages of onset in a family with a heterozygous *TSHR* variant, in which the proband with permanent CH and his father presented with hypothyroidism at birth and since the age of 30 years, respectively. This could be due to the defects in yet unidentified genes. Other possible causes of delayed diagnosis CH include residual activity of the gene product, iodine level status, and hypothalamic-pituitary-thyroid maturation (52–55).

The hyperthyroidism in the father of family 38 is difficult to explain based on what we know, and it may be a coincidental autoimmune phenocopy or the result of the modifying effect of other genetic variants.

We presented a range of extrathyroidal defects in some of our patients. The features observed in our studied families, including undescended testis and ophthalmic abnormalities, have also been reported in other studies in patients with CH in general and specifically in patients carrying different *TSHR* variants (12, 56). Some additional features in one family were possibly due to the co-inheritance of a *KAT6B* mutation. Accordingly, periodic examinations are recommended for children identified to have *TSHR* mutations, starting from the time of initial diagnosis, to monitor the clinical, hormonal, and intellectual outcomes of the patients. This could not only avoid serious consequences for patients but also provide genetic counseling and offer screening and preventive therapies to other family members and newborns.

The present study highlights the importance of genetic testing of families with CH and a positive history of non-autoimmune hypothyroidism, especially in the presence of extrathyroidal defects and variable phenotypes in the family, using high-throughput methods for a correct and deep insight into the genotype-phenotype correlation and to improve genetic counseling. However, further functional studies and investigations into the TSHR physiological functions and signal transduction might also help define the genotype-phenotype correlations for TSHR dysfunctions that have not yet been deciphered.

## Conclusion

The data from our study highlights the importance of considering the possibility of oligogenic inheritance in the CH pathogenesis that could lead to highly variable clinical phenotypes and different ages of onset in patients with CH in a family. Our study expands the *TSHR* variant spectrum and provides further evidence for the elucidation of the genetic etiology of CH and genotype-phenotype correlation. These findings emphasize the need for further research to clarify the molecular mechanisms underlying variable expressivity in CH.

## Data Availability

The original contributions presented in the study are publicly available. This data are available in the ClinVar repository with accession numbers as: SCV005911650- SCV005911651-SCV005911652- SCV005911653-SCV005911654-SCV005911655-SCV005911656.
